# Origin of adaptations to open environments and social behaviour in sabretoothed cats from the northeastern border of the Tibetan Plateau

**DOI:** 10.1098/rspb.2023.0019

**Published:** 2023-04-26

**Authors:** Qigao Jiangzuo, Lars Werdelin, Oscar Sanisidro, Rong Yang, Jiao Fu, Shijie Li, Shiqi Wang, Tao Deng

**Affiliations:** ^1^ Key Laboratory of Orogenic Belts and Crustal Evolution, School of Earth and Space Sciences, Peking University, 5 Yiheyuan Road, Beijing 100871, People's Republic of China; ^2^ Key Laboratory of Vertebrate Evolution and Human Origins of Chinese Academy of Sciences, Institute of Vertebrate Paleontology and Paleoanthropology, Chinese Academy of Sciences, Beijing 100871, People's Republic of China; ^3^ CAS Center for Excellence in Life and Paleoenvironment, Beijing 100044, People's Republic of China; ^4^ Division of Paleontology, American Museum of Natural History, New York, NY 10024-5102, USA; ^5^ Department of Palaeobiology, Swedish Museum of Natural History, Box 50007, S-104 05 Stockholm, Sweden; ^6^ Departamento de Ciencias de la Vida, Universidad de Alcalá, GloCEE -Global Change Ecology and Evolution Research Group, Alcalá de Henares 28801, Spain; ^7^ Hezheng Paleozoological Museum, Hezheng 731200, People's Republic of China; ^8^ University of Chinese Academy of Sciences, Beijing 100049, People's Republic of China

**Keywords:** *Machairodus*, *Amphimachairodus*, linxia basin, Eastern Asia, competition

## Abstract

The iconic sabretooth *Homotherium* is thought to have hunted cooperatively, but the origin of this behaviour and correlated morphological adaptations are largely unexplored. Here we report the most primitive species of *Amphimachairodus* (*Amphimachairodus hezhengensis* sp. nov.), a member of Machairodontini basal to *Homotherium*, from the Linxia Basin, northeastern border of the Tibetan Plateau (9.8–8.7 Ma). The long snout, laterally oriented and posteriorly located orbit of *Amphimachairodus* suggest a better ability to observe the surrounding environment, rather than targeting single prey, pointing to an adaptation to the open environment or social behaviour. A pathological forepaw of *Amphimachairodus* provides direct evidence of partner care. Our analyses of trait evolutionary rates support that traits correlated with killing behaviour and open environment adaptation evolved prior to other traits, suggesting that changes in hunting behaviour may be the major evolutionary driver in the early evolution of the lineage. *A. hezhengensis* represents one of the most important transitions in the evolution of Machairodontini, leading to adaptation in open environments and contributing to their further dispersal and radiation worldwide. This rapid morphological change is likely to be correlated with increasingly arid environments caused by the rise of the Tibetan Plateau, and competition from abundant large carnivores in this area.

## Highlights

The earliest *Amphimachairodus* discovered exhibits craniodental adaptation to open environment and social behaviour.

Adaptations to change in habitat and killing behaviour evolved prior to other traits.

Adaptatons to open environments and social behaviour first occurred near the Tibetan Plateau, probably due to aridification as the plateau was formed.

## 1. Introduction

The Plio-Pleistocene sabretoothed cat *Homotherium* represents a unique member of Felidae, with special hunting and social behaviours [1–6]. The origin of this behaviour and of correlated morphological traits remain largely unexplored, however, due to the paucity of well-preserved early representatives, and concomitant lack of targeted morphological studies.

The tribe Machairodontini, which *Homotherium* belongs to, can be traced back to the Miocene [7]. The Late Miocene represents the pinnacle of sabretoothed cat diversity [2,8,9]. The genus *Amphimachairodus* is the most widely distributed and successful genus among Late Miocene forms, and is known in Europe [10–12], western Asia [13], central Asia [14], eastern Asia [15–17], southeastern Asia [18,19] and North America [20–22]. This genus is mainly documented in the Turolian or deposits of equivalent age, with very abundant remains from the Linxia Basin (Yangjiashan Fauna and Qinpushan Fauna; 8–6 Ma [23]) and Baode in China (7.25–5.3 Ma [15,24]), and in the Quiburis Formation and Coffee Ranch in southwestern North America [25]. Many cranial traits of *Amphimachairodus* (e.g. the long snout and enlarged mastoid) are already *Homotherium*-like, suggesting potentially similar adaptations [7]. However, where *Amphimachairodus* originated and the function of these traits are still unclear.

**Figure 1.**
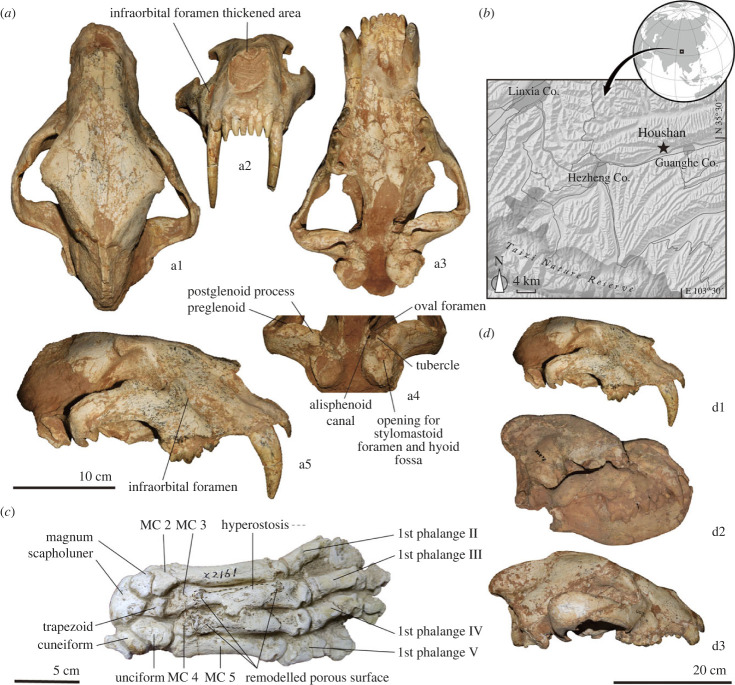
(*a*) Cranium of *Amphimachairodus hezhengensis* sp. nov. HMV2041. a1, dorsal view; a2, anterior view; a3, ventral view; a4, postero-ventral view; a5, lateral view. (*b*) Geography of fossil locality. (*c*) Pathology of the MC2 and MC3 of *Amphimachairodus* sp. HMV2047 forepaw. (*d*) Large predator contemporary with *A. hezhengensis* in the Linxia Basin. d1, *A. hezhengensis*, HMV2041; d2, *Dinocrocuta gigantea*, HMV2044; d3, Agriotheriini ursid, HMV2046.

In this study, we report a nearly complete cranium from Houshan locality, belonging to the Dashngou Fauna of the Linxia Basin (9.8–8.7 Ma), located on the northeastern border of the Tibetan Plateau [23,26] (figure 1). The cranium was listed in faunal list as *Machairodus palanderi* and *Amphimachairodus* sp. by previous authors but not formally reported or described [23,26]. The new cranium shows typical *Amphimachairodus* traits, representing the earliest member of the genus and providing a basis for investigating its peculiar morphology and adaptions.

## 2. Systematics

Order Carnivora Bowdich, 1821

Family Felidae Batsch, 1788

Subfamily Machairodontinae Gill, 1872

Tribe Machairodontini Gill, 1872

*Amphimachairodus* Kretzoi, 1929

Diagnosis: machairotont of large size. Rostrum long, and forehead wide. Orbit anterior border located at P4. Glenoid fossa overhung above basicranium. Mastoid process large, and paroccipital moderate to highly reduced. Mandibular flange weak or moderate, cornoid process small. Incisors large with serration, and upper I1 and I2 with laterally posited accessory cusps. P2 variably present. P3 with distinct anterior accessory cusp. P4 with distinct preparastyle and moderate to very small protocone. m1 with metaconid-talonid complex mostly absent.

Included species: *Amphimachairodus giganteus* (Wagner, 1848), *Amphimachairodus horribilis* (Schlosser, 1903), *Amphimachairodus palanderi* (Zdansky, 1924), *Amphimachairodus coloradensis* (Cook, 1922), *Amphimachairodus alvarezi* Ruiz-Ramoni *et al*. 2019 and *Amphimachairodus hezhengensis* sp. nov.

*Amphimachairodus hezhengensis* sp. nov.

*Machairodus palanderi* p.257, Deng *et al*. 2013

*Amphimachairodus* sp. p.11 Jiangzuo *et al*. 2023

*Holotype*: HMV2041, a nearly complete cranium (figure 1; electronic supplemenatry material, figures S1 and S2).

*Etymology*: After the place (Hezheng Paleozoological Museum, Hezheng, China) where the specimen was found and is currently stored.

*Type locality*: Houshan, Linxia Basin, Gansu province of northern China.

*Chronology and distribution*: Thus far only known from the early Late Miocene of northern China.

*Diagnosis*: medium-sized *Amphimachairodus* with small incisors and I1 with lingually posited accessory cusps; long C-P3 diastema; presence of P2; relatively small cheek teeth; small P4 preparastyle and moderate protocone.

*Differential diagnosis*: differs from *Machairodus* and *Nimravides* in having different cranial morphology, e.g. lower angle between facial and neurocranial part, wide forehead, retracted orbit and long rostrum, shorter and dorsally arched zygomatic arch, slightly overhanging glenoid fossa, more arched incisor row and more separated lingual accessory cusps in I2, presence of P2, more distinct P4 preparastyle and smaller protocone; differs from *Lokotunjailurus* in having larger size, longer C-P3 diastema, stronger P3 anterior accessory cusp and more robust P4; differs from other species of *Amphimachairodus* in having smaller incisors, I1 with two closely located lingual accessory cusps, longer C-P3 diastema, smaller cheek teeth, smaller P4 preparastyle and slightly larger protocone.

### (a) Description

See electronic supplementary material, appendix for detailed description and measurements.

## 3. Phylogenetic and biogeographic analyses

**Figure 2.**
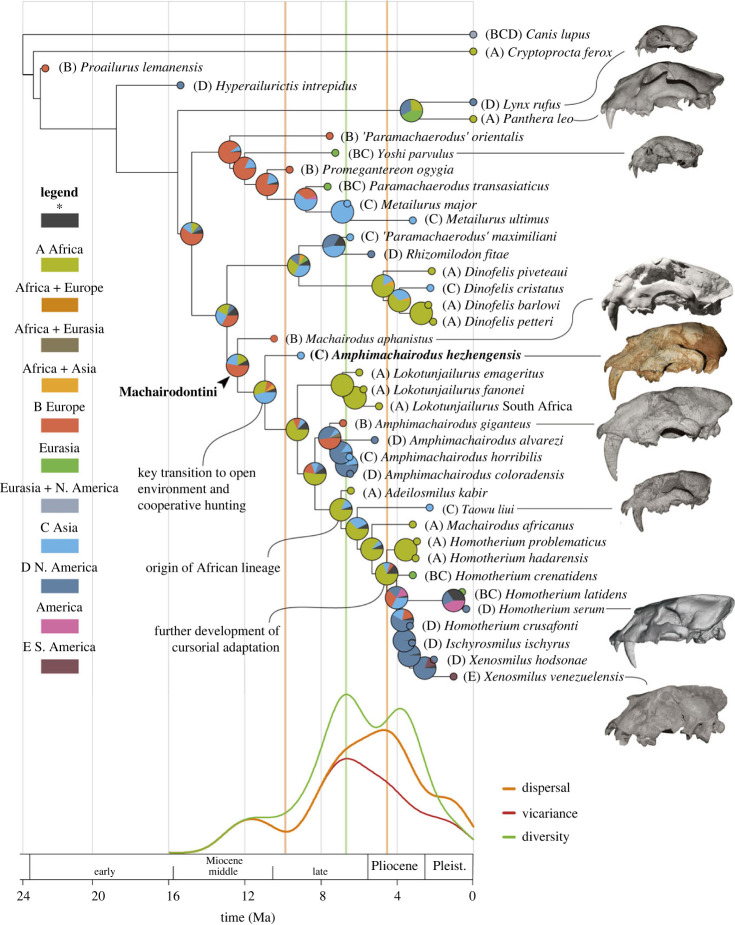
Tip-dating phylogeny of Machairodontinae. Biogeographic hypotheses (BioGeoBEARS implemented in RASP 4.2) are located at the nodes, with pie charts indicating the posterior probabilities of that node being present in a given geograpical region. The dispersal curves (for Machairodontini only, from *M. aphanistus* to Homotheriina) are located at the bottom of the figure. See the posterior probability of each node of the tip-dating phylogenetic analysis in the electronic supplementary material, appendix. The reconstruction of *Homotherium serum* from data in digimorph (http://www.digimorph.org/index.phtml). The photo of *Machairodus aphanistus* from Batallones comes from https://fossilhuntress.blogspot.com/2016/10/, the others are by the authors.

**Figure 5.**
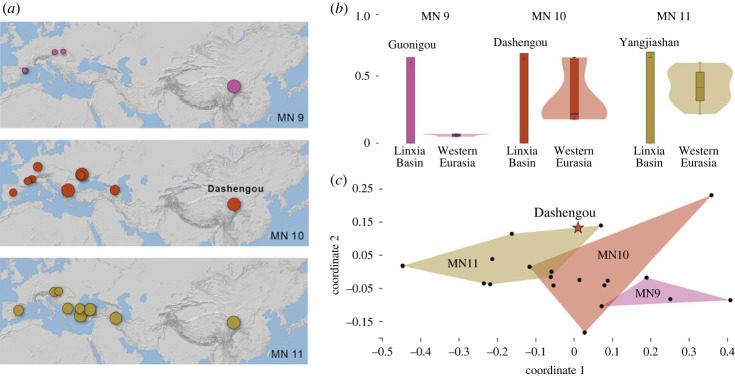
Composition analysis of the Linxia Basin fauna and contemporary faunae. (*a*) Proportion of open-adapted species in fauna from MN9-11 of Eurasia shown on the map, with the size of circle representing the proportion value (larger circle means more open species). (*b*) Proportion of open-adapted species in the faunas from MN9-11 of Eurasia. (*c*) Non-metric MDS analysis of faunal composition (family level diversity) of the Dashengou Fauna and faunae from western Eurasia.

The Bayesian phylogenetic analyses with and without tip-dating support that the new species is in a position intermediate between *Machairodus* and Turolian or equivalent-aged (herein ‘Turolian’) *Amphimachairodus* (figure 2; electronic supplementary material, figures S5 and S6). The Houshan cranium always forms the sister group of the lineage consisting of Turolian or equivalent-aged *Amphimachairodus*, *Lokotunjailurus*, *Taowu*, *Adeilosmilus* and Homotherina. The major differences lie in the different positions of *Lokotunjailurus*, which was sister to Homotheriina in ordinary bayes analysis (figure 5). The positions of metailurine felids are rather unstable in both two analyses and do not form monophyletic group in either analysis, but as our matrix mainly concerns the morphology of Machairodontini, the the position of metailurines in this study can be treated with circumspection.

Even though our analyses do not support that the Houshan cranium forms the sister group to a monophyletic *Amphimachairodus*, we do not erect a new genus for it as did Jiangzuo *et al*. [7] for *Adeilosmilus* and *Taowu*. This is because the Houshan material is close the Turolian *Amphimachairodus* in age and general morphology, and the potentially related genera *Lokotunjailurus* is only known from jaw fragmented and its ancestor form is largely unknown. The creation of a separate genus for the Houshan cranium is thus not justified and creates difficulty for assigning isolated material in the future. By contrast, *Adeilosmilus* clearly shows a craniodental morphology linking it to Homotherina, and *Taowu* is much younger and smaller, so the separation of these two genera is justified.

Our biogeographic analyses favour a BAYAREALIKE + J model, and imply that the lineage of *A. hezhengensis* and other more derived Machairodontini probably originated in Asia (figure 2). As seen in this figure, the diversification centre of Machairodontini changed several times in its evolutionary history. We further draw the diversification, dispersal and vicariance lines of this tribe, and find that the diversificaition and dispersal of this tribe increase dramatically after the appearance of *A. hezhengensis*. An adaptation to open environments (see below) and global aridification in the Late Miocene [27] contributed to its geographical expansion and diversification. Both diversification and vicariance reach their peaks in the latest Miocene, then reduce significantly following the great environmental change at the Miocene–Pliocene boundary [28–32], and only the African lineage (from *Adeilosmilus* to Homotheriina) survived. Subsequently, the dispersal of the tribe continues and reaches its peak in the Early Pliocene, resulting in a second increase in diversification. In the Pliocene, the Homotheriina evolved derived postcranial traits that are more adapted to open environments [33,34]. This adaptation increased the dispersal ability of Homotheriina, and weakens the vicariance effect, leading to the occurrence of widely distributed species (e.g. the pan-Eurasian *H. crenatidens* and *H. latidens*) but low diversity for the subtribe as a whole.

## 4. Evidence for adaptations to open environments and social behaviour in early Machairodontini

As in other species of *Amphimachairodus*, *A. hezhengensis* has a forehead that is much wider than the rostrum. A very wide forehead is seen in modern cheetah *Acinonyx jubatus* and snow leopard *Panthera uncia* and is correlated with an enlarged frontal sinus [35]. The frontal sinus is connected with the nasal passages through small ostia and is normally in continual communication with the nasal air [36]. An expanded frontal sinus can serve as a thermal buffer for cold hair and heat dissipation during running, and help in respiration. Both *Ac. jubatus* and *P. uncia* live in open environments [37]. The wide forehead in *Amphimachairodus* is therefore likely to be an adaptation to open environments. Such a wide forehead is absent in *Nimravides* and *Machairodus* [11,20,38]. Notably, *Homotherium* also has a wide forehead, whereas *Xenosmilus*, which has less cursorial postcranial bones and lived in forest, has a narrower forehead [39].

**Figure 3.**
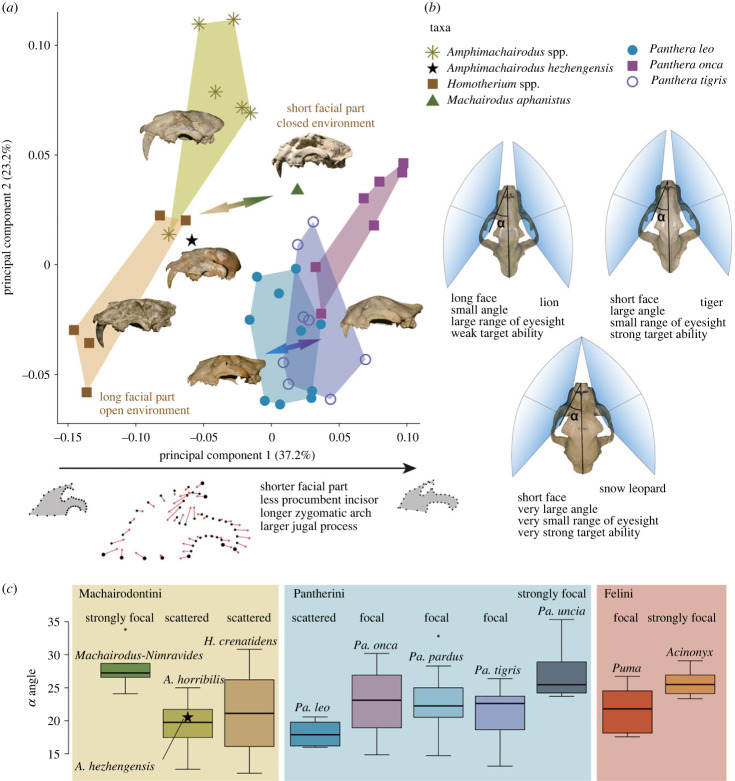
(*a*) Geometric morphometric analysis of cranial lateral profiles, showing the change in orbit position and length of the zygomatic arch along PC1. The arrow in the morphospace suggests similar patterns of difference between *Amphimachairodus*–*Machairodus* and *P. leo–P. tigris*. (*b*) Eyesight range and rostral length of some big cats, showing the different adaptations. (*c*) Boxplots of the angle between the long axis of orbit and the sagittal plane (in ventral view), showing the relationship between angle and focal ability.

The GM analysis (lateral view) also suggests that *A. hezhengensis* is located close to other species of *Amphimachairodus*, but is distinct from *Machairodus*. The major differences are in the first PC, which mainly explains differences in the length of the rostrum of the cranium and zygomatic length (figure 3*a*). It is interesting to note that the derived traits exhibited in *A. hezhengensis* are mainly cranial traits that are correlated with specialized hunting behaviour. The short zygomatic arch and large mastoid process suggest that masticatory strength is weakened, whereas the neck muscles tend to become better developed [3,40,41]. This suggests that the hunting behaviour of *A. hezhengensis* is closer to younger *Amphimachairodus* and *Homotherium*, in using a bite mainly effectuated by neck muscles to quickly kill the prey, and different from that of *Machairodus*, whose killing behaviour is probably effectuated mainly by the temporal and masseter muscles, like those of living big cats.

The long rostrum can lead to an increase in gape. The less angled facial and neurocranial parts, retracted orbit (in the middle of the cranium), and long rostrum are reminiscent of the modern lion *Panthera leo*. This morphology may be related to a wider field of view but a weaker ability to target a single point. We calculated the angle between the long axis of the orbit and the sagittal plane (in dorsal view; figure 3*b* for details). Even though there is overlap, the lion *Pa. leo* has a significantly smaller angle compared with other species of *Panthera*, which otherwise have similar angles, except *Pa. uncia* with a very large angle (figure 3*c*). A small angle suggests a laterally oriented orbit. In Felini, *Puma* has a similar angle to that of most species of *Panthera*, whereas *Acinonyx* is close to *Pa. uncia*. *Machairodus* and *Nimravides* have large angles, similar to *Pa. uncia* and *Acinonyx*, whereas *Amphimachairodus* and *Homotherium* have similar values to *Pa. leo*. Of this feature, *A. hezhengensis* is within the range of variation of *A. horribilis*, which has a similarly laterally oriented orbit. In open environments, prey are more conspicuous, and thus precise targeting by stereo vision becomes less necessary in general, but *Acinonyx* is able to run swiftly when chasing prey, and *P. uncia* needs to target caprine prey in rock cover. Both species require stereo vision. A laterally oriented and posteriorly located orbit on the side of the cranium allows for a wider field of view (figure 3*b*), and assists in prey identification, as well as targeting other companions during cooperative hunting, a trait more correlated with social behaviour. This adaptation is well developed in lions as compared to other pantherine cats and is developed to a still greater degree in *Amphimachairodus*. The situation in *Machairodus* is more similar to that of the tiger (figure 3*a*,*c*). Such contrasts suggest similar differences in adaptation between *Amphimachairodus* and *Machairodus*, just like lion and tiger.

Our ancestral state reconstruction (electronic supplementary material, figure S9) supports that this unique cranial trait first evolved in *A. hezhengensis* and remains unchanged in more derived Machairodontini. The difference in angle between *Amphimachairodus* and that of the cheetah suggests that the hunting behaviour of *Amphimachairodus* is closer to *Pa. leo* than to *Ac. jubatus*.

**Figure 4.**
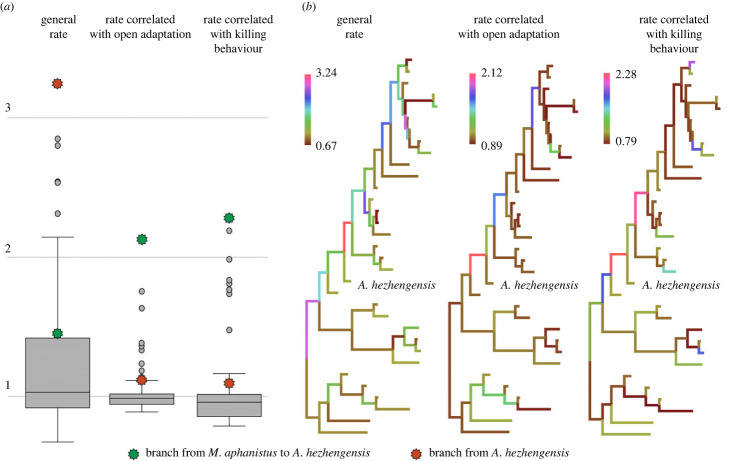
Evolutionary rates of traits of different blocks. (*a*) Boxplots of rates among the three blocks and (*b*) rates of different blocks of each branch.

Our analyses of the evolutionary rate of traits support that the traits correlated with open environment, as well as those correlated with killing behaviour (see detailed set in Material and Methods section), exhibit the highest rates in the branch between *M. aphanistus* and *A. hezhengensis* (figure 4). This suggests that the evolution from *Machairodus* to *Amphimachairodus* that occurred in the Linxia Basin represents the most significant adaptative transition in Machairodontini evolution. The general traits exhibit the highest rate from *A. hezhengensis* to more derived Machairodontini, and many of these involve cheek teeth. The cheek teeth of *A. hezhengensis* are small and less derived, suggesting that the cutting efficiency of *A. hezhengensis* is weaker than that of the Turolian or equivalent-aged species of *Amphimachairodus*. The increased cheek tooth size from *A. hezhengensis* to the derived species of *Amphimachairodus* reflects enhanced cutting efficiency for shortening foraging time, possibly in the face of increased diversity of scavengers, including hyaenids, at that time.

A pathological forepaw (figure 1*c*) is also known from the Linxia Basin (unclear locality). The forepaw is intact, with carpals, MC2-5, and corresponding phalange preserved. The size (MC3 length 101.86 mm) is similar to a small tiger or lion (101.01–124.00 mm, *n* = 17) and is similar in morphology. The middle phalanges are asymmetric to accommodate the claws. The claw is very large, and shows a gradually smaller size from the second claw to the fifth claw. This morphology fits with *Amphimachairodus*. The MC3 and MC4 became fused during the healing, which greatly restricted their normal function for prey capture and is likely to reduce down the running speed of the individual. Such an injury would severely influence the hunting success of the animal, yet the remodelled porous surface in MC3 and MC4 indicates the development of a chronic condition during healing, suggesting that the individual continued to survive after injury for quite a long time. The healed fractures support the existence of partner care, pointing to social behaviour in *Amphimachairodus*.

## 5. The origin of *Amphimachairodus* from the northeastern border of the Tibetan plateau

The discovery from the middle Bahean (equivalent to MN10 of Europe) makes *A. hezhengensis* the earliest known definite *Amphimachairodus*. In Europe, a very large collection of *M. aphanistus* is found in Batallones 1 and 3, Spain, both MN10 [11,38]. *A. hezhengensis* is distinctly more derived than *M. aphanistus* from these Spanish sites. This is in agreement with our biogeographic analyses, implying *Amphimachairodus* originated on the northeastern border of the Tibetan Plateau.

As seen in the analyses above, the cranial morphology of *Amphimachairodus* shows adaptations to open environments and cooperative hunting. This coincides with the paleoenvironmental evidence. The significant rise of the northern Tibetan Plateau in the Middle and Late Miocene [42] has a huge impact on the ecosystem of the Linxia Basin, which experienced a significant faunal restructuring at the Middle/Late Miocene boundary, and almost none of the Middle Miocene genera continue into the Late Miocene, and nearly all mammals from the Guonigou Fauna are new immigrants [43]. These new immigrants are represented by large high-crowned elasmotherines and hipparionine horses, both pointing to an open and dry environment. This environment continues to the Dashengou fauna, and hipparionines in this fauna also show more cursorial adaptations than contemporary hipparionines in Europe [44]. By contrast, there is no significant faunal change at the Middle/Late Miocene boundary in Europe, and many Middle Miocene genera continue into the early Vallesian [45].

To further test this hypothesis, we compiled the faunal list of the three faunas—Guonigou, Dashengou and Yangjiashan—with ages equivalent to MN9, MN10 and MN11 of Europe. We also selected 23 western Eurasian faunas from MN9–MN11 with abundant remains. These were downloaded from the NOW database [46] with amendments from the literature (see electronic supplementary material, appendix for details). We identify each species to open or closed environment, judging from dental morphology and previously published microwear for herbivores [47–49], and craniodental traits of carnivores (see electronic supplementary material, appendix for details). We found a high proportion of species with open environment adaptations in the earliest Late Miocene in the Linxia Basin and these environments persisted in the basin (figure 4). By contrast, western Eurasia shows a gradual increase in aridification since MN9 and shows geographical heterogeneity as previous research has suggested [50,51]. The overall faunal composition (at the family level) of the Dashengou Fauna is similar to MN11 faunas of western Eurasia as revealed by a non-metric MDS analysis, supporting the early aridification of the Linxia Basin. In the more eastern part of China in the lower Bahe Formation, the faunal is still dominant by forest species, e.g. viverrid, suid, cervid and low-crowned bovid [52], suggesting that at the beginning of the late Miocene, the arid area is still restricted to Tibetan Plateau at this time.

The early arid environment in the Linxia Basin, probably due to the uplift of the Tibetan Plateau to a significant height at this time [53,54], provides an environmental trigger for the appearance of open-environmental adaptions among mammals. Open environments also benefit social behaviour [55,56], due to the increasing visibility of carnivores to each other, and also of preys, which makes them more difficult to catch. When such arid environments spread at the MN11, *Amphimachairodus* followed.

**Figure 6.**
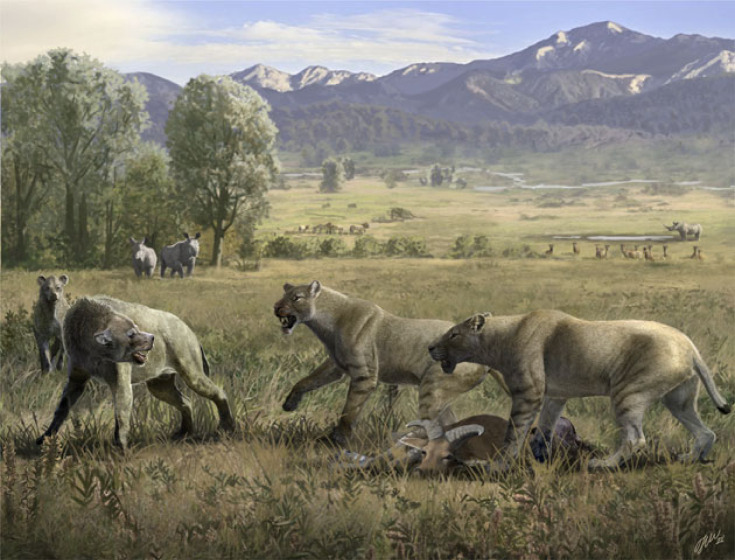
Reconstruction of two *Amphimachairodus hezhengensis* defending their prey (*Hezhengia bohlini*) from two *Dinocrocuta gigantea*. Artwork by Oscar Sanisidro.

Another factor that pushed the rapid morphological evolution and social behaviour of *Amphimachairodus* is the presence of abundant large Carnivora on the northeastern border of the Tibetan Plateau. These are especially represented by the huge bone-cracking percrocutid hyaena *Dinocrocuta gigantea* [57]. This hyena is by far the most common species of Carnivora in the Dashengou Fauna and reaches a body mass estimate of approximately 380 kg based on dental size [58], though the known postcranial bones [59], and an unpublished skeleton from the Linxia Basin suggests it is not that large, but similar to *Amphimachairodus* in size (see a life reconstruction in figure 6). Two unpublished agriotherine bears (size of *Indarctos atticus*) are also present in this fauna. Such abundant large predators not only compete with machairodonts, but also bring direct threats to their lives, as large carnivore tends to kill the cubs or juveniles, or single individual of other carnivores, just as modern spotted hyena and lion [60]. The open environment increases such competition and threats due to limited cover and high visibility. In Europe, *Machairodus* of the Vallesian often coexisted with *Indarctos* and amphicyonids with similar body size to that of *Machairodus* [61–64], and these species are often less abundant than *Machairodus* [11], but in the Dashengou Fauna of the Linxia Basin, *Dinocrocuta* is much more abundant than *Amphimachairodus* [26]. The defense for territory and life from other carnivores is an important trigger of social behaviour in Carnivora [65]. The Quaternary hyaenids *Pachycrocuta* is known as prey kleptoparasite of sabretoothed cats [66]. This is probably also the case in the Linxia Basin for *Dinocrocuta* and *Amphimachairodus*, and the two large agriotherine bears probably also have a bone-cracking diet and involve in such behaviour. The abundant large predators/scavengers on the northeastern border of the Tibetan Plateau may have created significant pressure on the early evolution of Machairodontini (figure 6).

## 6. Material and methods

### (a) Institutional abbreviations

**AMNH FM** fossil mammal collection of the American Museum of Natural History, New York, USA

**AMNH F:AM** Frick collection (fossil mammals), Division of Paleontology, AMNH, New York, USA

**HM(V)** Hezheng Paleozoological Museum, Hezheng, China

**IVPP** Institute of Vertebrate Paleontology and Paleoanthropology, Chinese Academy of Sciences, Beijing, China

**UCMP** University of California Museum of Paleontology, Berkeley, USA

**YLSNHM** Yingliang Stone Natural History Museum, Quanzhou, China

### (b) Other abbreviations

**BI** Bayes inference

**H** height

**L** length

**M/m** upper/lower molar

**MN** units of the Neogene land mammals of Europe

**MP** maximum parsimony

**P/p** upper/lower premolar

**OTU** operational taxonomic units

### (c) Fossils and methods

The material of the new taxon described in this study is housed at the Hezheng Paleozoological Museum, Hezheng, China. Material of *Machairodus* at the AMNH, material of *Amphimachairodus* at the AMNH, HM, IVPP, UCMP, YLSNHM, and material of *Homotherium* from AMNH, IVPP and HM, were examined for systematic study.

Terminology of skull anatomy follows Qiu *et al*. [67], with minor modifications. The measurements follow Jiangzuo & Liu [68] with modification, and are shown here in electronic supplementary material, figure S1. Parts of the figure plots were made in the software package ggplot2 [69] in R [70].

Bayesian inference using MrBayes 3.2.7 [71,72] was employed in the phylogenetic analyses. We performed both a non-dating method and a tip-dating method with a relaxed clock model [73]. In practice, Smilodontini has an unstable position and causes long-branch attraction, but this it is not the focus in this study, so we excluded this tribe in the analyses. Constraint on Machairodontinae was both performed in MrBayes.

Our matrix is adopted from Jiangzuo *et al*.[7], adding the new species and two corresponding traits: the antero-posterior width of the glenoid fossa, and shortening of the zygomatic arch (in red in electronic supplementary material, appendix).

For analyses of the evolutionary rate of different characters, we subdivided 71 traits into three categories: (i) general traits, including traits 9–45, 48, 57, 59–63, 65–67 and 70; (ii) traits correlated with open environments, including traits 50–54 and 58; (iii) traits correlated with killing behaviour, mainly those describing incisor size and canine morphology, and some key craniomandibular traits (e.g. length of the zygomatic arch, size of the mastoid process and size of the coronoid process), including traits 1–8, 46–47, 49, 55–56, 64, 68–69 and 71. The analyses were based on the tip-dating tree.

For biogeographic analyses, we tested different models using the methods proposed in BiogeoBEARS [74] implemented in the software RASP 4.2 [75,76]. The detailed setting of geographical information is provided in the electronic supplementary material, appendix. The best-fit model was chosen by weighted AICc. The diversity, dispersal and vicariance lines of Machairodontini are drawn through time from 12 Ma to present. The ancestral state was reconstructed using Bayestraits implemented in the software RASP 4.2 [75,76].

### (d) Fossil locality

Houshan is a classical fossil site within the Linxia Basin, belonging to the Dashengou Fauna, best known for producing hundreds of skulls of *Hezhengia* [77]. Very abundant mammalian remains have been found from this fauna (see details in [23]). The huge hyaenid *Dinocrocuta gigantea* is the most abundant carnivore. The machairodont in this fauna was previously determined as *Machairodus palanderi* [23], and a juvenile mandible was recently determined as *Machairodus aphanistus* [78]. Other carnivores known include two medium-sized Agriotheriini bears, three mustelids (*Promeles* sp., *Martes* sp. and *Pekania* sp.), two skunks (*Promephitis parvus* and *Promephitis hootoni*), one barbourofelid (*Albanosmilus* sp.) and four hyaenids, the latter being *Hyaenictitherium* sp. (smaller than typical *H. wongii*), *Ictitherium* cf. *viverrinum*, *Adcrocuta eximia* and a new small form with a short rostrum. An age estimate of 8.7–9.8 Ma is proposed for the fauna [23].

## Data Availability

Data used in this study can be found in electronic supplementary material, appendix [79].
